# Tailored Approach in Adrenal Surgery: Retroperitoneoscopic Partial Adrenalectomy

**DOI:** 10.3389/fendo.2022.855326

**Published:** 2022-03-28

**Authors:** Pier Francesco Alesina, Polina Knyazeva, Jakob Hinrichs, Martin K. Walz

**Affiliations:** ^1^ Clinic for Endocrine Surgery, Helios Universitätsklinikum Wuppertal, Wuppertal, Germany; ^2^ Department of Surgery and Centre of Minimally Invasive Surgery, Evang. Kliniken Essen-Mitte, Essen, Germany

**Keywords:** adrenalectomy, partial adrenalectomy, minimally invasive, adrenal surgery, conn, pheochromocytoma, cushing adenoma

## Abstract

The interest on partial adrenalectomy has steadily increased over the past twenty years. Adrenal pathologies are mostly benign, making an organ-preserving procedure attractive for many patients. The introduction of minimally invasive techniques played probably an important role in this process because they transformed a complex surgical procedure, related to the difficult access to the retroperitoneal space, into a simple operation improving the accessibility to this organ. In this review we summarize the role of partial retroperitoneoscopic adrenalectomy over the years and the current indications and technique.

## Introduction

The first description of experimental partial adrenalectomy is attributable to Frederick Gates. In 1918, he investigated the potential role of the adrenal glands in antibody formation (in guinea pigs) and found out that these glands are not an essential part in immunity processes. Moreover, they observed that the removal of three-quarters to seven-eighths of the adrenal tissue does not cause adrenal insufficiency (1). The first description of partial adrenalectomy in humans was published in 1934 based on the hypothesis that medullary adrenal hyperplasia is responsible for severe arterial hypertension and bilateral partial adrenalectomy might be its therapy (2). The operation that consisted in the removal of two-third to three-fourth of each adrenal was performed in two stages from the back through a “so-called” kidney incision under spinal anesthesia. This report includes 8 cases with no mortality or severe complications. The first report of partial adrenalectomy for bilateral adrenal tumors was published in 1982 but the operation was performed in 1965 (3). A 13-year-old boy underwent total adrenalectomy on the right side and partial on the left side for bilateral pheochromocytoma. For two days after surgery the patient received diminishing doses of cortisol and 6 days postoperatively 5 mg prednisone was given twice a day orally. The substitution therapy was discontinued after 4 months and a computer tomography scan 15 years later proved the adrenal remnant without tumor recurrence. In 1983, Irvin and colleagues, and in 1984 van Heerden and coworkers reported on partial adrenalectomy in bilateral pheochromocytomas each in 3 patients (4, 5). Irvin described a family of a father and two daughters with bilateral pheochromocytomas who had been recurrence-free for 3–8 years after partial adrenalectomy. The Mayo group mentioned “enucleation” of bilateral pheochromocytomas in two patients in the early 1950s and a third case of an airline pilot (5) published more in detail as a case report in 1985 (6). By successful function-preserving adrenalectomy on both sides the latter patient could continue his professional career. As none of these patients—mainly with hereditary diseases—developed a tumor recurrence, the concept of partial adrenalectomy seemed to be a real alternative to life-long corticoid replacement with high risks of Addisionian crisis and death (7, 8) and became an alternative strategy for selected patients in adrenal surgery. The first reports of minimally invasive partial adrenalectomy were published by us in 1996 (9). In 5 cases (2 Conn’s adenomas, 3 non-functioning tumors) the retroperitoneoscopic approach was used.

## Material and Methods

A literature search (Medline database) with the keywords “partial adrenalectomy” and/or “cortical-sparing adrenalectomy” and “retroperitoneoscopic partial adrenalectomy” has been performed and the data were analyzed. The present review and the search from the literature focused specifically on the retroperitoneoscopic technique. Papers with >10 reported cases have been included while smaller series and case reports were not considered for the present analysis. Additionally, all the data presented have been extracted from our previous publications with a specific focus on partial adrenalectomy performed by the retroperitoneoscopic approach (10–16). **Table 1** summarizes the results of the studies presented in this review.

**Table 1:** Retroperitoneoscopic partial adrenalectomy.

Author	Year	Disease	No. of Patients	No. of partial adrenalectomies	Follow-up (months)	Comments
Walz et al. (10)	2006	Pheochromocytoma	127	57	45	In 21 of 22 patients with bilateral adrenal pheochromocytomas, partial adrenalectomy was performed at least on one side
Alesina et al. (14)	2012	Pheochromocytoma	66	89	48	The study included only patients with bilateral disease
Walz et al. (16)	2018	Pheochromocytoma	31	31	109	children and adolescents between 7 and 20 years old, 18 had unilateral and 13 bilateral disease
Sasagawa et al. (17)	2003	Conn’s syndrome	47	13	Not reported	All patients with functional cortical adenoma and pheochromocytoma showed normal adrenal function after the operation
Walz et al. (12)	2008	Conn’s syndrome	183	47	59	No difference in the rate of blood pressure improvement was found in patients with partial adrenalectomy versus those with total adrenalectomy
Fu et al. (18)	2011	Conn’s syndrome	212	104	96	Patients in the partial adrenalectomy group showed similar improvement in hypertension compared to total adrenalectomy (n = 108)
Alesina et al. (13)	2010	Cushing’s syndrome	170	44	71	No recurrence
Lowery at al (15).	2017	Cushing’s syndrome	42	35	40	Only patients with bilateral disease were included; remission rate 92%
He et al. (19)	2012	Cushing’s syndrome	93	87	Length not indicated	Recurrent or persistent hypercortisolism was observed after surgery in one patient
Xu et al. (20)	2015	Non-functioning adenomas, concomitant hypertension	75	69	24	Hypertension cured in 35% of the patients and improved in 31%, no recurrence
Zhang et al. (21)	2007	Cysts	14	14	12	Nine cases of cyst decortication and five cases of partial adrenalectomy; no recurrence

## The Surgical Technique

**Figure 1:**
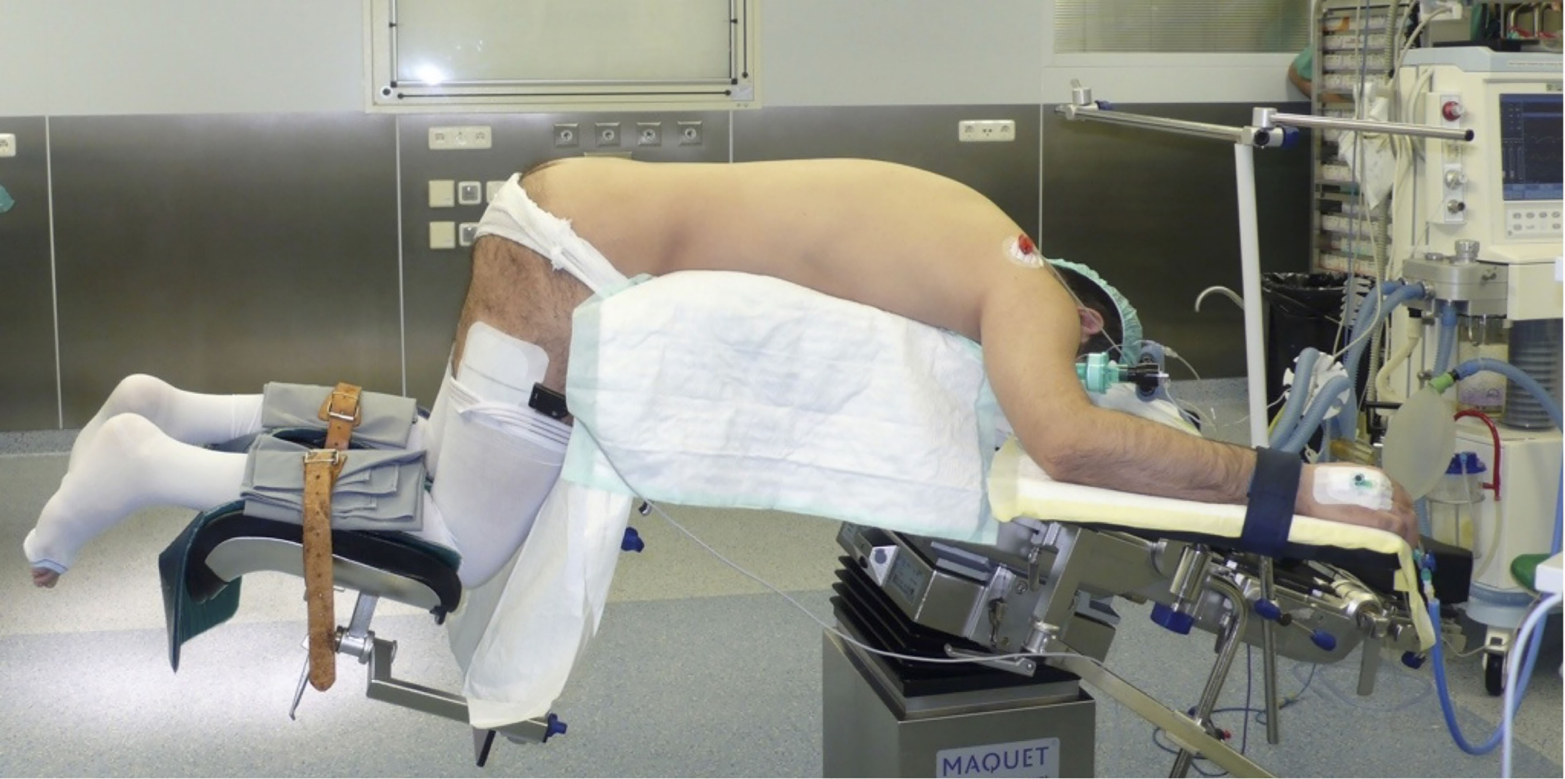
Patient’s position.

**Figure 2:**
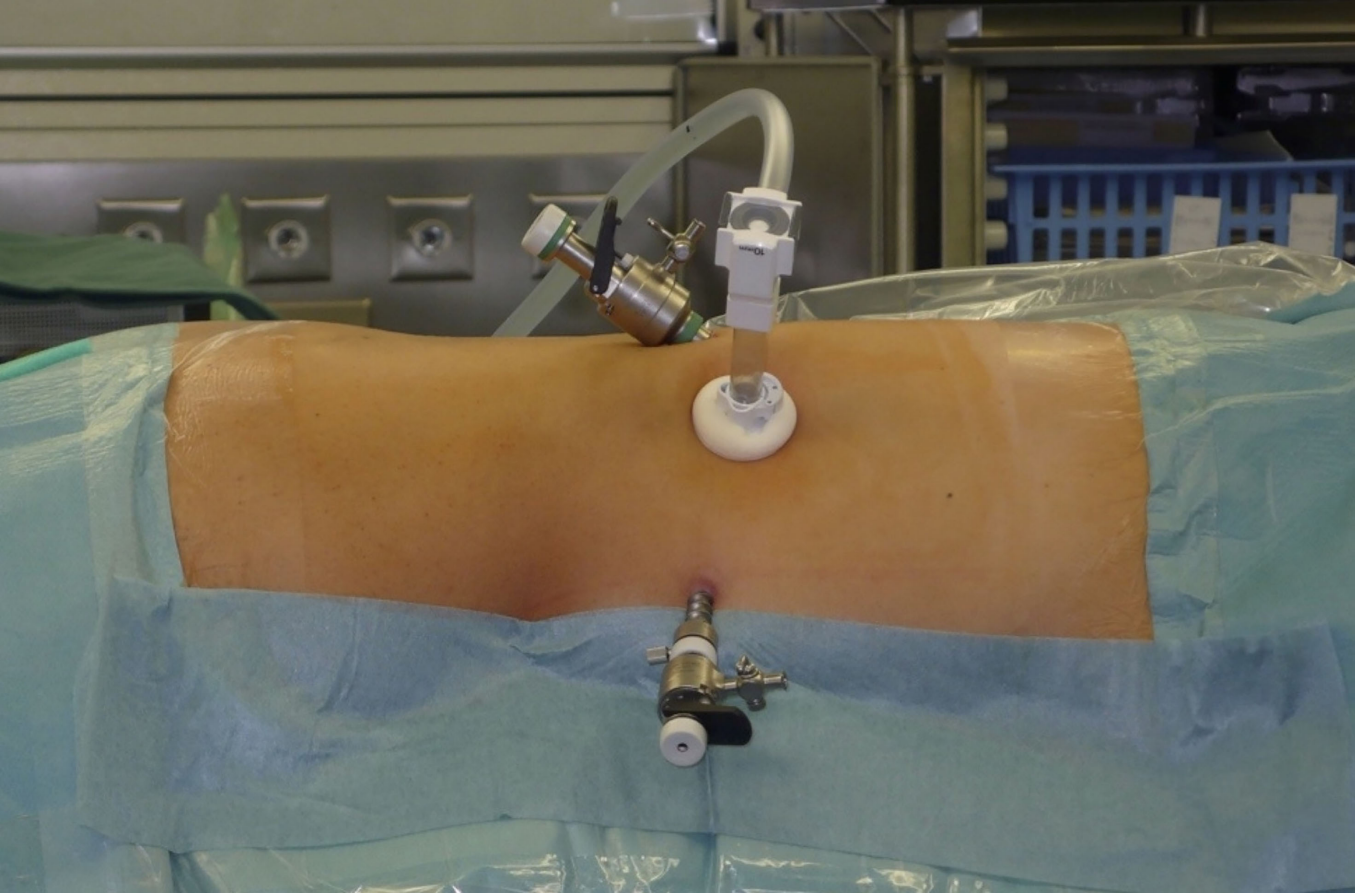
Trocar’s position.

**Figure 3:**
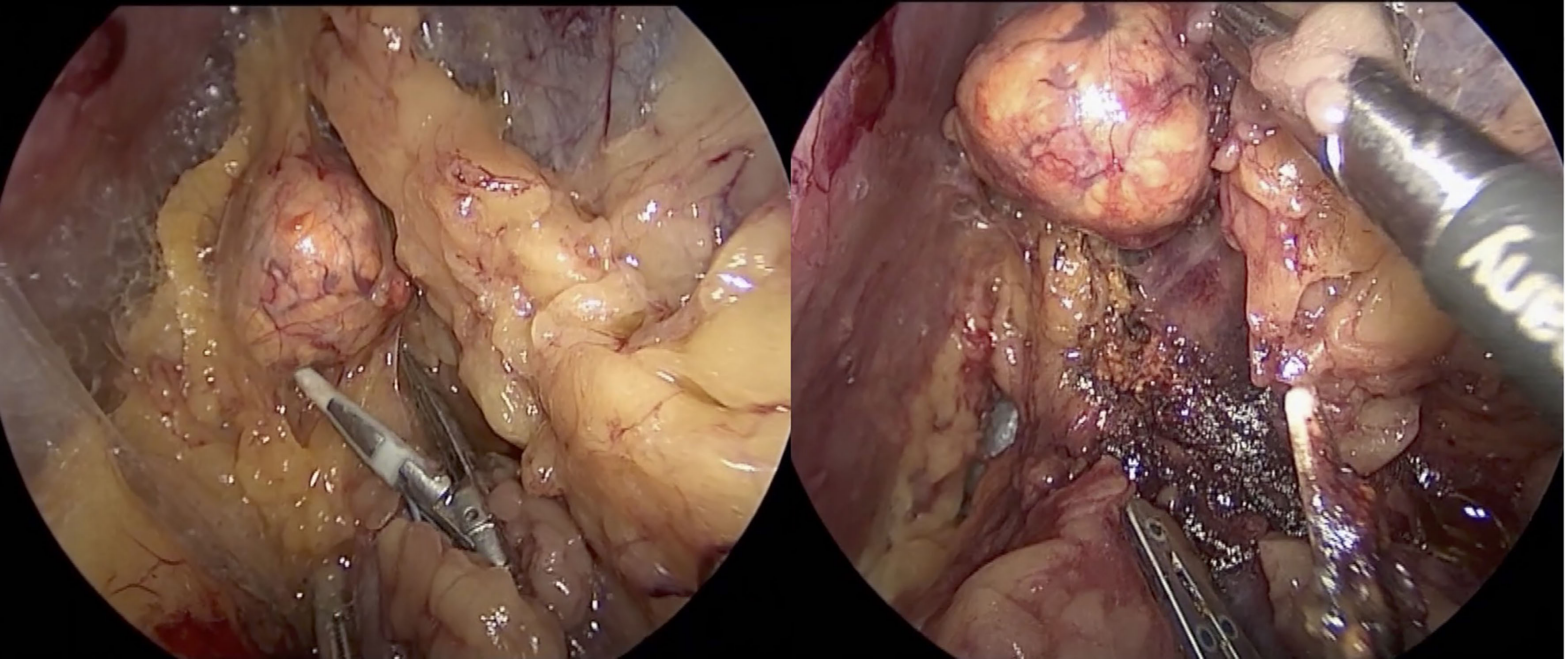
Tumor located on the upper pole on the right side: the dissection of the lower part of the adrenal gland is avoided.

**Figure 4:**
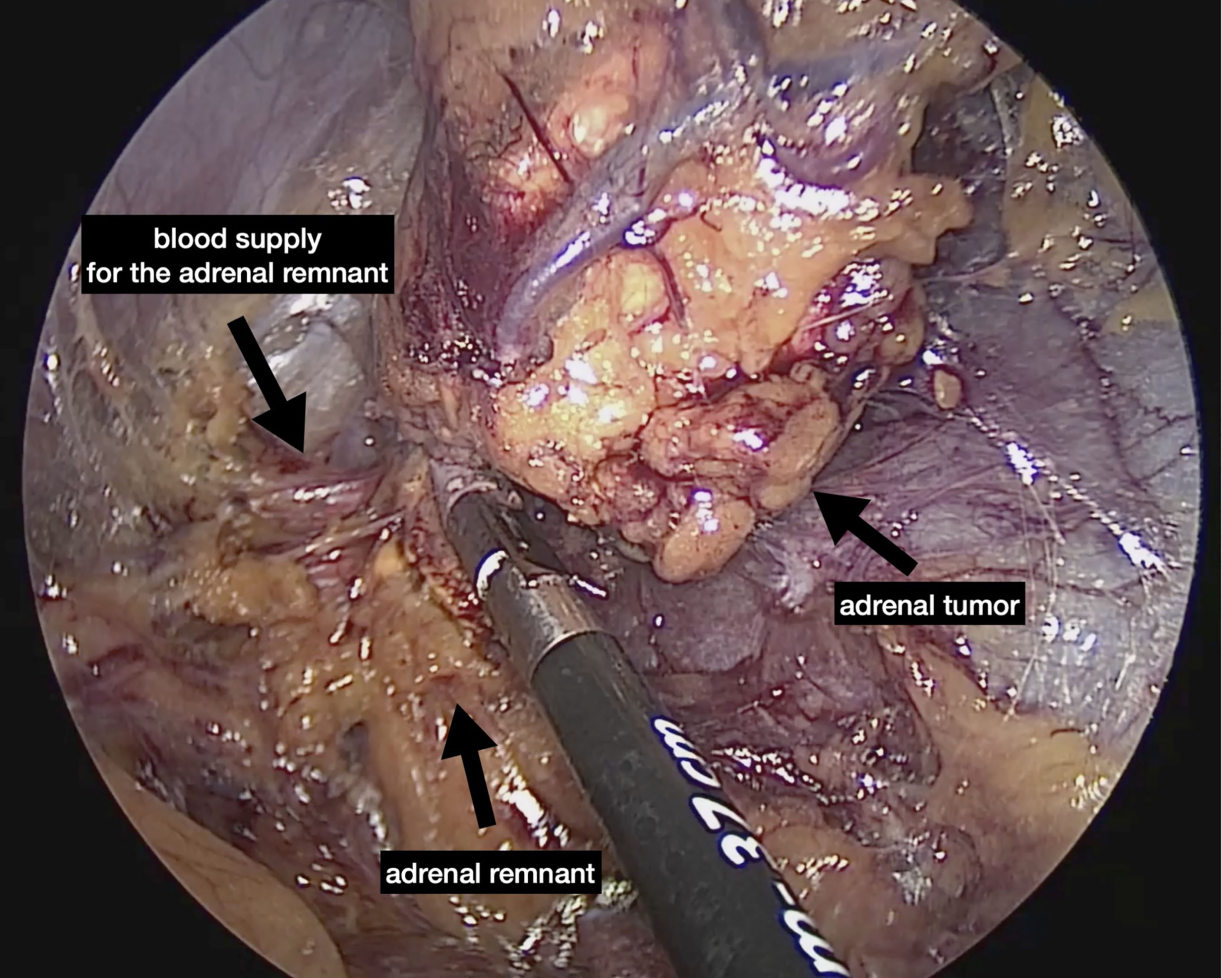
Division of the gland performed by bipolar instrument.

The first description of an experimental retroperitoneal endoscopic adrenalectomy was published in 1993 by Brunt et al. (22). The authors performed the procedure in a domestic swine model using insufflation of the retroperitoneal space with carbon dioxide and concluded that the posterior route could have been potentially suitable to the treatment of adrenal lesions. In 1994 and 1995 retroperitoneal adrenalectomy in humans has been described in Japan, New Zeeland, Sweden, Italy, Germany and Turkey (23–28) including our own paper (29). Some used the lateral approach (23–25, 27), others the posterior access (26–29). The latter is more accepted today with the patient in the prone, half-jackknife position. On our own hands a rectangular pillow is used that allows the abdominal wall to hang through ventrally (**Figure 1**). Alternatively, a roll may be positioned below the chest and the pelvis. The angle of the hip joint should be around 90°. This position creates an optimal space between the ribs and the iliac crest. A 1.5 to 2 cm skin incision is performed at the level of the 12th rib and the retroperitoneal space is reached by blunt and sharp dissection with scissors. A finger is inserted into the retroperitoneum in order to position a 5 mm port inserted just below the tip of the 11th rib under digital control. A blunt trocar with an inflatable balloon and an adjustable sleeve (Medtronic, Minneapolis, USA, ®) is introduced into the initial incision site and blocked. Under CO_2_ insufflation pressure of 20–30 mmHg a working space is created by opening Gerota’s facia and pushing all retroperitoneal fatty tissue ventrally. By this maneuver the region around the kidney and the adrenal gland are visualized. A third trocar (5 or 10 mm in diameter) is inserted under visual control paying attention to avoid the subcostal nerve running parallel to the 12th rib. The final position of the trocar is demonstrated in **Figure 2**. The dissection starts on the upper pole of the kidney that is freed from the adhesions to the retroperitoneal fatty tissue. The kidney is gently retracted caudally and medially to expose the lower pole of the adrenal gland. The dissection is then continued medially from caudal to cranial. On the right side the vena cava is visualized and the retrocaval dissection is performed until the adrenal vein is reached; on the left side the adrenal vein is isolated medially when completing the dissection of the lower pole of the adrenal. The cranial dissection represents the last step of the procedure. Total adrenalectomy is usually performed as an “en-bloc” resection of the gland and retroperitoneal fatty tissue and needs to be modified in partial adrenalectomy. Preconditions for successful function-preserving adrenal surgery are special knowledges in anatomy and surgical technique. Adrenal glands are perfused from medial, inferior, and cranial but not from lateral. By this, adrenal tissue can be dissected in any direction preserving at least one direction of perfusion. The preservation of the main vein is not mandatory. Planning of cortical-sparing procedures is based on a detailed analysis of preoperative imaging in order to understand position and size of the tumor within the gland. Intraoperative ultrasound may facilitate the identification of neoplasias, especially in obese patients. For tumors located at the upper pole of the gland, the dissection of the lower pole should be avoided and vice versa (**Figure 3**). The division of the adrenal tissue can be performed by any energy device and residual bleeding from the remnant that can be easily controlled by standard coagulation instruments (**Figure 4**).

In recent years indocyanine green (ICG) fluorescence has been used to visualize viability of adrenal remnant after resection. The first report was published in 2013 by Manny and colleagues in three patients during robotic adrenalectomy (30). In the setting of a collaboration with the Institute for Research Against Cancer of the Digestive System (IRCAD) in Strasbourg we analyzed the behavior of the adrenal tissue in a porcine model and found out that fluorescence imaging can provide real-time guidance during minimally invasive adrenal surgery. Prior to dissection, it allows to easily discriminate the adrenal gland from surrounding retroperitoneal structures. After adrenal gland division, ICG injection associated with a computer-assisted quantitative analysis helps to distinguish between well-perfused and ischemic segments (31). These findings have been confirmed in some studies analyzing different adrenal pathologies in the humans (32, 33). Fluorescence guidance can be also useful to estimate more precisely the volume of the adrenal remnant. This is particularly important in patients submitted to bilateral surgery to decide about the necessity of corticosteroids substitution. It is well-known that preservation of 15 to 30% of one gland is necessary to avoid a substitution therapy (34). Brauckhoff et al. studied 10 patients with bilateral adrenal tumors which underwent measurement of plasma adrenocorticotropic hormone (ACTH), serum cortisol, and maximal cortisol liberation with an ACTH test after subtotal bilateral adrenalectomy, which left 15 to 30% of adrenal tissue in situ. In the early postoperative period, all patients had normal basal serum cortisol levels, despite in 6 patients a pathologic ACTH test result was observed. During follow-up all patients were found to have a normal ACTH test result. None of the patients required long-term steroid supplementation. This is confirmed by our experience on 66 patients operated for bilateral pheochromocytomas (14). All patients with preservation of less than 15% of adrenal tissue ultimately became steroid-dependent.

### Pheochromocytoma

Partial adrenalectomy should always be considered in the case of pheocromocytoma. The risk of developing bilateral tumors is much higher than historically assumed, as part of the well-known 10%-rule for pheochromocytoma. This is particularly true when considering that familial diseases account nowadays for almost 40% of the cases (35). The main consequence of performing adrenal preservation, especially in hereditary pheochromocytoma, is the possible development of recurrent disease that needs to be weighed against avoidance of a lifelong steroid therapy. Nevertheless, a clear distinction between ipsilateral and contralateral recurrence should be made, as only ipsilateral recurrence can be avoided if total instead of partial adrenalectomy is undertaken. Older studies based on open partial adrenalectomy reported recurrence in up to 20% (35), whereas recent papers using minimally invasive techniques describe lower recurrence rates of less than 10%. Data of the European-American-Asian-Bilateral-Pheochromocytoma-Registry including a total of 625 patients with bilateral tumors show that partial adrenalectomy was performed for smaller tumors compared to total adrenalectomy (3 vs. 3.5 cm) and more often since 2010 (36). This seems to be related to the increased use of minimally invasive techniques which allow a more precise dissection. Recurrent ipsilateral pheochromocytoma developed in 35 of 625 patients (5.6%); 33 out of 248 patients (13%) after partial adrenalectomy and 2 out of 301 (0.6%) after total adrenalectomy. Moreover, metastatic pheochromocytoma was diagnosed in 8 of 625 patients (1.3%). Our group published the results of partial adrenalectomy for pheochromocytoma in 2006 (10). In this series 94 unilateral and 12 bilateral tumors were removed by the posterior approach. Fifty-seven partial adrenalectomies had been performed. There was neither mortality nor conversion to open surgery. A bleeding occurred after left-sided resection in a patient with bilateral pheochromocytomas. This patient required transfusion of 4 units of blood and retroperitoneoscopy to remove the hematoma on the first postoperative day. After a mean follow-up of 45 ± 33 months no recurrence was observed. Later on, we reported the results in the subgroup of children and adolescents between the age of 7 and 20 years (16). There were 35 retroperitoneoscopic and two combined laparoscopic–retroperitoneoscopic operations. Thirty-one partial adrenalectomies have been performed. None of the bilateral pheochromocytoma patients needed corticoid supplementation following partial adrenalectomy. After a mean follow-up of 9.1 ± 4.6 years, 2 patients affected by von Hippel–Lindau disease developed an ipsilateral recurrent after 16 and 22 months, respectively. These tumors were removed by redo surgery by the retroperitoneoscopic approach.

According to the data available, the risks of recurrence and malignancy are low and justify, in our opinion, the use of partial adrenalectomy in most cases. Moreover, redo surgery by the posterior retroperitoneoscopic approach is almost always feasible independent of the surgical access of the first operation (11). Partial adrenalectomy can achieve lifelong steroid independency in most of the patients affected by bilateral tumors. We published the results of surgery on a group of 66 patients treated for bilateral disease (14). Fifty-seven patients (88%) were affected by genetic diseases. In 32 cases surgery was synchronously performed on both sides, in 34 cases unilateral adrenalectomy followed previous surgery on the contralateral side. A cortical-sparing resection was possible in 89 procedures resulting in a corticoid-free postoperative course in 60 patients (91%). A postoperative corticosteroid substitution therapy was necessary in six patients. After a median follow-up period of 48 months, one patient showed a persistent disease and needed reoperation, none developed a recurrent disease. Partial adrenalectomy is suggested also from the Endocrine Society guidelines (37) at least for patients with hereditary pheochromocytoma, with small tumors who have already undergone a contralateral complete adrenalectomy. The recommendation is based on the low risk of recurrence reported (7% over 3 years, and 10–15% over 10 years) and the high probability of steroid-independency (78–90%).

### Conn’s Syndrome

Preliminary results of partial adrenalectomy for hyperaldosteronism performed by the retroperitoneoscopic route have been reported by our group in 1998 (38). Between 1994 and 1997, 11 out of 22 patients which underwent cortical-sparing surgery were affected by Conn’s adenomas. These tumors are generally small and have a negligible risk of malignancy, therefore partial adrenalectomy seems to be a good alternative to total adrenalectomy. In 2003 Sasagawa and colleagues reported the results of partial adrenalectomy performed in 47 cases including 13 cases of Conn’s adenomas (17). There was one conversion to open surgery because of bleeding (2%). The authors described as intraoperative complications three (6.4%) adrenal bleedings, two (4.3%) pneumothoraces, one (2.1%) massive hemorrhage (more than 1,000 ml) and one (2.1%) injury of the renal vein. In 2008 we reported the results of partial adrenalectomy in 47 patients which represented 26% of those operated for Conn’s syndrome at our Institution between August 1994 and January 2007 (12). After a mean follow-up of almost 5 years, completed for 160 patients (87%), the rate of patients with improvement of hypertension was similar following partial (n = 37) and total adrenalectomy (n = 123): (92% vs. 85%, p = 0.41). Our results are confirmed by a prospective randomized study performed on 212 patients (108 and 104 who underwent total and partial adrenalectomy, respectively) (18). Intraoperative blood loss in the partial adrenalectomy group was significantly higher than in the total adrenalectomy group (p <0.05) but no patient needed blood transfusion. Patients in both groups showed improvement in hypertension, and in all aldosterone returned to normal.

Opponents of cortical-sparing surgery are concerned about the possible presence of multiple nodules or adrenal hyperplasia in the tumor-carrying gland. The decision to perform partial adrenalectomy should be weighed against the risk of leaving a remnant with pathological tissue. In our series from the 2008 histologic examination revealed solitary adenomas in 127 patients and nodular hyperplasia in 56. Patients with an adenoma were significantly younger than those with nodular hyperplasia (47.8 ± 13.1 vs. 53.8 ± 10.9 years, p <0.05). Suitable candidates for cortical-sparing surgery are particularly young patients (<45 years old) with a clearly visible nodule on preoperative imaging. In most of the series reported in the literature the cure rate of cortical-sparing surgery is even better than after total adrenalectomy probably because of this selection bias (39). Moreover, a recent German multicentric study showed that both, postoperative hypocortisolism (11.5% vs 25.0% after partial and total adrenalectomy, respectively; p <0.001) and postoperative hypoglycemia (2.6% vs 7.1%; p = 0.039) occurred more frequently after total adrenalectomy. No recurrence was encountered in both groups (40).

## Cushing’s Syndrome

The first report on partial adrenalectomy for hypercortisolism has been published in 1934 by Walters (41). In this study ACTH-dependent and independent cases are included and a total number of 46 partial adrenalectomies with a mortality rate of 15% is reported. Certainly, partial adrenalectomy is nowadays not an option for ACTH-depending Cushing’s syndrome, as recurrences are obligatory. In cases of ACTH-independent hypercortisolism caused by adrenal adenomas or bilateral macronodular hyperplasia cortical-sparing surgery can be considered. In 2010 we published the results of retroperitoneoscopic adrenalectomy for clinical and subclinical Cushing’s syndrome. In this series 157 patients suffered from unilateral adrenal disease and 13 patients from bilateral macronodular hyperplasia. There were 44 partial adrenalectomies performed with no ipsilateral recurrence after a mean follow-up of 70.9 months (13). There are three different strategies in case of bilateral hyperplasia causing hypercortisolism: bilateral total adrenalectomy, unilateral adrenalectomy guided by the size of the glands or result of adrenal venous sampling, bilateral surgery including cortical-sparing adrenalectomy at least on one side. Unilateral surgery and bilateral surgery with partial adrenalectomy can both avoid substitution therapy. We reported the results of bilateral surgery on 42 patients with clinical or subclinical Cushing’s syndrome (15). Thirty-nine out 42 patients were treated by cortical-sparing surgery, namely, unilateral resection (n = 3), unilateral adrenalectomy (n = 15), bilateral resection (n = 9), adrenalectomy and contralateral resection (n = 14). After median follow-up of 40 months the remission rate was 92%; 11 patients required ongoing steroid supplementation. There were three biochemical recurrences after unilateral surgery (two underwent contralateral resection); two patients with new/progressive radiological nodularity were biochemically eucortisolaemic at the time of last follow-up. He and colleagues reported on the results of adrenal-sparing surgery in 87 cases (31 performed by open surgery, 56 by retroperitoneal laparoscopy) (19). The cure rate was 97.8%; recurrent or persistent hypercortisolism was observed after surgery in one patient in which total adrenalectomy was performed. Despite the increasing evidence published in the literature, partial adrenalectomy is still not recommended for patients with hypercortisolism in any of the published guidelines. Nevertheless, the evidence from the literature allow to consider it especially for tumors smaller than 4 cm which harbor a negligible risk of malignancy.

## Non-Functioning Tumors

Adrenal non-functioning incidentalomas have a 2 to 5% chance of malignancy. The likelihood of a benign adrenal tumor is higher in the group of adrenal incidentalomas ≤6 cm (42). A study from Xu et al. analyzed the effect of adrenal surgery on blood pressure in patients with non-functioning adrenal adenoma and concomitant hypertension (20). After a complete endocrinological evaluation confirming the hormonal inactivity of the tumor 77 out 186 patients underwent surgery according to the diameter of the tumor (>4cm) or preference of the patient. Retroperitoneoscopic partial adrenalectomy was performed in 69 patients, and six patients underwent retroperitoneoscopic total adrenalectomy while two patients had open total adrenalectomy. After two years of follow-up 27 patients (35%) in the surgery group were cured from hypertension, whereas 24 (31%) improved, and 26 (34%) remained refractory when compared with the control group. Therefore, the authors conclude that early partial adrenalectomy can play a role in patients with incidentalomas and concomitant hypertension. Preoperative imaging could determine the risk for malignancy and define the indication for surgery which is based on the size of the tumor. As surgery is usually performed to exclude malignancy, partial adrenalectomy is not routinely recommended for those patients. Moreover, cortical-sparing surgery is mostly not feasible for tumors larger than 6 cm because of the absence of normal adrenal tissue. Conversely, it could be performed in individually selected cases for tumors between 4 and 6 cm with a low malignant potential and in case of bilateral tumors. Adrenal symptomatic cysts requiring resection are an ideal indication for partial adrenalectomy. The largest series of cortical-sparing surgery for adrenal cysts has been published by Zhang et al. (21) which performed a cortical-sparing procedure in 14 cases. The retroperitoneoscopic resection was performed with the patients in the lateral decubitus.

## Metastasis

Partial adrenalectomy is not a standard procedure for adrenal metastasis. It can be indicated in selected cases in patients after previous adrenalectomy on the contralateral side or in case of bilateral metastasis. Few case reports have been published mostly on adrenal metastasis from renal cell carcinoma (43–45).

## Conclusions

Over the last twenty years partial adrenalectomy has become the preferred operation for patients with bilateral pheochromocytoma and patients with metachronous tumors after previous total adrenalectomy on the contralateral side. Moreover, it is a recognized option in case of unilateral pheochromocytoma and Conn’s adenoma. The role of cortical-sparing surgery is uncertain for patients with Cushing’s syndrome and incidentaloma, as there are only few studies in the literature investigating the long-term results of partial adrenalectomy in this subgroup of patients.

## Author Contributions

PFA and PK wrote the manuscript. JH contributed to the discussion. MKW participated in the design of this study and edited the manuscript. All authors listed have made a substantial, direct, and intellectual contribution to the work and approved it for publication.

## Conflict of Interest

The authors declare that the research was conducted in the absence of any commercial or financial relationships that could be construed as a potential conflict of interest.

## Publisher’s Note

All claims expressed in this article are solely those of the authors and do not necessarily represent those of their affiliated organizations, or those of the publisher, the editors and the reviewers. Any product that may be evaluated in this article, or claim that may be made by its manufacturer, is not guaranteed or endorsed by the publisher.
